# Characterization of LED‐based hybrid diffuse reflectance spectroscopy method for determination of SPF and UVA‐PF in blinded multi‐centre study (ALT‐SPF)

**DOI:** 10.1111/ics.70007

**Published:** 2025-09-01

**Authors:** Carina Reble, Michael Bayer, Bertrand Colson, Tanja Emmler, Jean‐Claude Hubaud, Matthias Seise, Eva Perroux‐David, Georg Wiora, Daniela F. Zamudio Diaz, ALT‐SPF Consortium, Martina C. Meinke, Stephan Bielfeldt

**Affiliations:** ^1^ Courage + Khazaka electronic GmbH Cologne Germany; ^2^ Charité — Universitätsmedizin Berlin, Department of Dermatology, Venerology and Allergology Berlin Germany; ^3^ Dermatest GmbH Muenster Germany; ^4^ QuoData GmbH Dresden Germany; ^5^ Helioscience Marseille France; ^6^ SGS Proderm GmbH Schenefeld/Hamburg Germany

**Keywords:** claim substantiation, emulsions, spectroscopy, statistics, sun protection factor, ultraviolet‐A protection factor

## Abstract

**Objective:**

The consortium ALT‐SPF performed an international round robin test to characterize non‐invasive methods as alternatives to the erythema‐based testing of sun protection factor (SPF) according to ISO 24444:2019.

**Methods:**

Hybrid diffuse reflectance spectroscopy (HDRS) based on a multi‐lambda LED light source uses in vivo reflectance spectra on skin to determine sunscreens in vivo absorbance spectra, which are fused with respective in vitro absorbance spectra measured as thin films transmission as described in ISO 24443:2019. As a part of the ALT‐SPF consortium initiative, a blinded study on 64 samples was performed in four European laboratories. After further improvements of the method, a blinded re‐evaluation based on 16 samples was performed. Five statistical acceptance criteria for new methods were assessed by an independent statistical institute to compare the obtained results to the reference methods for SPF and UVA‐PF.

**Results:**

The initial ALT‐SPF study 1 showed that the bias criterion was acceptable, while the reproducibility and interlaboratory variability needed further improvement. The re‐evaluation study 2 showed that the reproducibility and interlaboratory variability could be considerably improved. Using only *n* = 10 volunteers and a bias correction based on the initial ALT‐SPF study 1 data, the SPF results of the re‐evaluation study 2 were close to the acceptance criteria of the ALT‐SPF study with criterion 1 (reproducibility) and only 11% over the limit defined by the performance of the gold standard. The UVA‐PF results were within the acceptance limits for the acceptance criteria, except criterion 3 being in the ‘almost met’ range. The re‐evaluation study indicates that the method has a comparable precision to the gold standard methods ISO 24444 and ISO 24443.

**Conclusion:**

This study showed that the LED‐HDRS method is capable of providing reasonable non‐invasive SPF and UVA‐PF results and that the performance shows close alignment to the reference method. Thus, it can be proposed as an alternative method to the current standards ISO 24444 and ISO 24443.

## Graphical abstract

The LED‐HDRS method applies a multi‐lambda LED light source and spectrally resolved detection for diffuse reflectance spectroscopy of skin with and without sun protection. The performance of the method was characterized in the ALT‐SPF study.

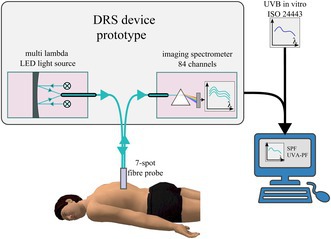

## Résumé

**Objectif:**

le consortium ALT‐SPF a réalisé un test interlaboratoire international pour caractériser les méthodes non invasives pouvant remplacer les tests basés sur l'érythème pour déterminer l'indice de protection solaire (SPF) conformément à la norme ISO 24444:2019.

**Méthodes:**

la spectroscopie hybride de réflectance diffuse (HDRS), basée sur une source lumineuse à LED multi‐lambda, utilise les spectres de réflectance in vivo sur la peau pour déterminer les spectres d'absorbance in vivo des écrans solaires, qui sont fusionnés avec les spectres d'absorbance in vitro correspondants, mesurés sous forme de transmission de films minces comme décrit dans la norme ISO 24443:2019. Dans le cadre de l'initiative du consortium ALT‐SPF, une étude en aveugle portant sur 64 échantillons a été réalisée dans quatre laboratoires européens. Après avoir apporté des améliorations supplémentaires à la méthode, une nouvelle évaluation en aveugle a été effectuée sur 16 échantillons. Cinq critères d'acceptation statistique pour les nouvelles méthodes ont été évalués par un institut statistique indépendant afin de comparer les résultats obtenus aux méthodes de référence pour le SPF et l'UVA‐PF.

**Résultats:**

l'étude initiale ALT‐SPF 1 a montré que le critère de biais était acceptable, tandis que la reproductibilité et la variabilité interlaboratoire nécessitaient une amélioration supplémentaire. L'étude de réévaluation 2 a montré que la reproductibilité et la variabilité interlaboratoire pouvaient encore être considérablement améliorées. En utilisant uniquement *n* = 10 volontaires et une correction du biais basée sur les données de l'étude 1 ALT‐SPF initiale, les résultats relatifs au SPF de létude de réévaluation 2 étaient proches des critères dacceptation de létude ALT‐SPF avec le critère 1 (reproductibilité) et seulement 11% au‐dessus de la limite définie par la performance de la norme de référence. Les résultats relatifs à l'UVA‐PF se situaient dans les limites d'acceptation pour les critères d'acceptation, à l'exception du critère 3 qui se situait dans la fourchette « presque atteint ». L'étude de réévaluation indique que la méthode présente une précision comparable à celle des méthodes de référence ISO 24444 et ISO 24443.

**Conclusion:**

cette étude a montré que la méthode LED‐HDRS était capable de fournir des résultats relatifs au SPF et à l'UVA‐PF non invasifs raisonnables et que ses performances étaient très proches de celles des méthodes de référence. Elle peut donc être proposée comme méthode alternative aux normes actuelles ISO 24444 et 24443.

## INTRODUCTION

With the development of UV light emitting diodes (LEDs), new application fields opened up due to the unique properties of these light sources that are tunability and fast electrical switching, small size and low heat production. Replacement of other light sources in established dermatological applications has already been proposed [1, 2], and applications like skin disinfection [3, 4] have opened new fields of research.

Diffuse reflectance spectroscopy (DRS) has been used for many years for skin measurements in the different spectral regions, including sun protection research [5, 6, 7, 8, 9, 10]. Within the ‘Advanced UV For Life’ consortium, which had the aim to facilitate development and application of UV‐LEDs, a research project was initiated in 2015, to develop a non‐invasive method for sun protection factor (SPF) determination based on LEDs and DRS. To be able to measure sunscreens with high SPFs, the hybrid DRS principle (HDRS) was implemented. It combines in vivo DRS measurements in the UVA range with additional transmission spectroscopic measurements with polymethylmethacrylate (PMMA) plates to also determine the UVB protection of the assessed sunscreen products. The method was first published by Ruvolo et al. [6] and has recently been standardized and published as ISO 23698 [11]. Yet, this ISO standard is specified only for Xenon arc light sources and the two optical systems described therein.

The first study using a multi‐LED spectroscopic HDRS setup (with 1st generation demonstrators) showed that SPF and UVA‐protection factor (UVA‐PF) values obtained by this new approach correlated well with the ISO reference values using 11 market products as well as customized research sunscreen products [12]. In addition, two additional test institutes performed measurements with the sunscreen standards of different ISO norms. Using a process‐specific calibration based on the first study, the results on standard sunscreens matched expectations [13].

Subsequently, four laboratories with improved LED‐HDRS devices participated in the international ALT‐SPF ring study, which is described in the main article of this special issue [14]. The aim of this ring study was to characterize the method performance of alternative SPF and UVA‐PF methods and to compare its results to the ISO reference methods 24443:2021 [15] and 24444:2019 [16]. A set of 64 blinded sunscreen samples was investigated with a sophisticated study design and statistical evaluation method, which is described by Colson et al. [14]. Based on the learnings from the large amount of empirical data obtained, further investigations and a second re‐evaluation ring study with four test laboratories were performed.

The aim of this publication is to summarise and review the results obtained by the LED‐HDRS method in the ALT‐SPF study and to evaluate its potential to function as a non‐invasive alternative to the classical methods of SPF and UVA‐PF measurements.

## MATERIALS AND METHODS

### Samples

The 32 (64 due to duplicates) sunscreen samples were chosen by the ALT‐SPF consortium to be a representative selection of market products, as described in the ALT‐SPF method paper [14]. The products were divided into 8 product groups (PG). The product groups contained emulsions in the range of SPF 5 (PG 2), SPF 15 (PG 3), SPF 30 (PG 4) and SPF 50/50+ (PG 5) for medium viscosity, for SPF 30 with low (PG 1) and high viscosity (PG 6), and one‐phase ethanol sprays (PG 7) with SPF 50/50+ as well as products with high mineral content (PG 8) with SPF 50/50+. Each product group consisted of 4 products with duplicates for each sample.

For the re‐evaluation study 2, 16 samples included 4 duplicates of PG1 (SPF30, low viscosity), a selection of one sample of PG2 (product 2–3), PG3 (product 3–3), PG4 (product 4–2), and PG8 (product 8–2) plus the standard sunscreens P5, P6, P8, and S2.

All products were blinded by QuoData for all participants (test centres and operators) and unblinded by QuoData after receiving the results.

### ALT‐SPF factorial study design (example for product group 1 of 8 groups)

The factorial study design is shown in Table 1. Due to the study design, results of blinded duplicates were obtained in independent settings (combinations of 3 factors). Each panel consisted of *n* = 5 volunteers. The complete design and statistics of the ALT‐SPF study are described in the main paper of Colson et al. [14].

**TABLE 1:** Exemplary factorial design for one product group applied to all eight product groups in ALT‐SPF (study 1) and PG 1 of re‐evaluation (study 2).

Test product	Volunteer panel (*n* = 5)	In vivo technician	In vitro technician
LE_LC01_11	1	1	1
LE_LC01_12	2	2	2
LE_LC01_13	1	2	1
LE_LC01_14	2	1	2
LE_LC01_15	1	2	2
LE_LC01_16	2	1	1
LE_LC01_17	1	1	2
LE_LC01_18	2	2	1

### Validation criteria

Data obtained with the alternative methods were compared with the ALT‐SPF reference data measured according to ISO 24444:2019. The complete study design and statistical evaluation is described in Ref. 14. The statistical validation criteria are described in Table 2.

**TABLE 2:** Description of statistical criteria applied in the ALT‐SPF study.

Criterion 1	Reproducibility precision	Reproducibility standard deviation $s_{R}$ must be smaller than of reference method
Criterion 2	laboratory bias	Laboratory standard deviation $s_{L,\text{pers}}$ must be smaller 0.3 ln SPF
Criterion 3	Reproducibility precision + variation of bias across products within product group	Augmented $\;s_{R}$ should be smaller than $s_{R}$ of the reference method
Criterion 4	product group mean bias	Alternative method bias must be smaller than descision limit (DL), < 0.4 ln SPF for PG 2
Criterion 5	variation of product group bias across product groups	s must be less than consitency limit (CL)

### DRS device

The aim of the chosen optical setup was not only to use LEDs but also to employ a spatially and spectrally resolved detection in order to be able to capture inhomogeneities prior to algorithmic averaging. The technical principle of LED‐based DRS has been described elsewhere [12]. In short, LED‐HDRS is based on DRS in a customized system using multiple UVA LEDs with peak wavelengths at 325 nm, 340 nm, 355 nm, 365 nm, 380 nm, and 390 nm, which are coupled into an illumination fibre bundle. While previous versions of the setup [12] included UVB LEDs as well, beginning with the ALT‐SPF study, additional UVA LEDs (at 325 nm and 340 nm) were implemented in the device instead of UVB LEDs in order to increase the UVA power in the wavelength range of hybridization. An imaging spectrometer (HORIBA Jobin Yvon GmbH, Oberursel, Germany) is used for spectrally resolved detection. A customized fibre bundle based on 100 μm fibres with 7 subunits (each transmitting all illumination wavelengths) and two rings of detection fibres in each subunit is used for skin illumination and detection of diffusely reflected light. A spatial offset between the central illumination fibres and the detection fibres avoids detection of direct reflections, which do not pass the sunscreen layer.

For each exposure, test measurements with 30 ms (and if necessary 300 ms) are automatically performed in order to ensure optimal use of dynamic range while avoiding detector saturation. The erythema effective dose of the measurement was below 13 J/m^2^, using a maximum irradiation time of 4.3 s (4.0 s exposure plus test exposure measurement). This corresponds to <0.13 standard erythema dose (SED).

The erythema effective dose is calculated based on the measured LED spectra of the device, including minor UVB contributions due to the spectral width of LED spectra (typically 14 nm full width at half maximum for the 325 nm LED).

The total measurement time includes the irradiation time, plus two dark measurements without LED irradiation, used to ensure a correct subtraction of background signals.

A simple model of light propagation through the sunscreen layer, the underlying skin and a second time through the sunscreen layer leads to the equation

(1) $$T\left(\lambda \right)=\sqrt{\frac{R}{R_{0}}}$$

where $T\left(\lambda \right)$ is the transmission spectrum, R is the diffuse reflectance after product application and $R_{0}$ the diffuse reflectance before product application. However, a calibration of the system needs to account for the influence of fibre optics design as well as the skin optical properties, which determine the pathlength of detected photons. The calibration was derived from previous data by a fit of
(2)

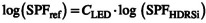

with *C*_*LED*_ being a scalar fit factor, which corresponds to

(3) $${\mathrm{SPF}}_{\mathrm{ref}}={\left({\mathrm{SPF}}_{\text{HDRSi}}\right)}^{C_{\mathrm{LED}}}$$

In order to align with ISO 23698, describing the correction of HDRS results based on device‐dependent multiplication factor, *C*_cal_ can be described by

(4) $$C_{\mathrm{Cal}}=\frac{{{\mathrm{SPF}}_{\text{HDRSi}}}^{C_{\mathrm{LED}}}}{{\mathrm{SPF}}_{\text{HDRSi}}}\cdot e^{b}$$

with *b* being the bias correction applied in results obtained after the ALT‐SPF study 1; see Table 3 for details.

**TABLE 3:** Overview of ALT‐SPF (study 1) and re‐evaluation (study 2).

	ATL‐SPF study 1	Re‐evaluation study 2
Number of samples	64	16
Product groups (PG)	1, 2, 3, 4, 5, 6, 7, 8 (4 samples with 2 duplicates for each PG)	1 (4 samples with 2 duplicates), 2, 3, 4, 8 (one sample of each PG) P5, P6, P8, S2 (standard sunscreens)
Number of labs	4	4
Volunteers per panel	5	10
Hybridization range	320–330 nm (original data) 320–345 nm (recalculations)	320–345 nm
Results described in	Figures 2 and 3, Tables 4, 5, 6, 7 (original data), Tables 8 and 9 (recalculations)	Figures 6 and 7, Tables 10, 11, 12, 13
Calibration factor	*C*_Cal_ = 0.8695 (original data in Figures 2 and 3, Tables 4, 5, 6, 7) *C*_Cal_ = 0.8695 (original data in Tables 8 and 9 plus bias correction with *b* = 0.164) *C*_Cal_ = 0.9174 (recalculations in Tables 8 and 9 with photodegradation as in ISO 23698 plus bias correction with *b* = 0.164)	0.9174 (with photodegradation as in ISO 23698 plus bias correction with *b* = 0.164)
Photodegradation correction	Linear with T (Figures 2 and 3, Tables 4, 5, 6, 7) as in ISO 23698 (Tables 8 and 9, recalculations)	as in ISO 23698

### In vivo measurements

The in vivo measurements presented in this paper were carried out at different time points, starting with the ALT‐SPF study, additional measurements for lab comparisons, and the follow‐up re‐evaluation study. Detailed study conditions are described in Table 3.

These studies were completed in accordance with the principal requirements of the declaration of Helsinki and according to the main principles of Good Clinical Practice (GCP). Healthy volunteers (between 18 and 70 years old) were informed both orally and in writing of the study details including potential risks and inconveniences. They provided their written consent before they were included in the study. The study protocol was approved by the ethics committee of the Charité Universitätsmedizin Berlin (EA1/268/21). Measurements were performed in four labs with LED–HDRS devices.

Exclusion criteria were: Age below 18 or over 70 years, inability to make independent decisions, pregnant and lactating women, individual typological angle (ITA) < 28°, medication with photosensitizing potential, known skin disease, previous abnormal reaction to UV light, stains, blemishes, or pigments within the measurement area, sun‐damaged skin in the measurement area (e.g. strong wrinkles or diffuse pigmentation), known allergies or intolerances to cosmetic products, strong hair on the back at the time of the measurement (hair may be shaved 3 days before the measurement or can be clipped on the day of the measurement).

All measurements were performed on the volunteers' back skin. Skin pigmentation was measured colourimetrically (CR‐400 Chroma Meter, Konica Minolta, Inc., Tokyo, Japan or Skin‐Colorimeter CL 400, Courage + Khazaka electronic GmbH, Cologne, Germany) using ITA° as a parameter. The location of ITA° measurements coincided with the sunscreen application areas (test sites). In order to ensure sufficient skin homogeneity, the ITA° differences within the measurement fields were not larger than 5°. The panel average of ITA° values was aimed to be in the range of 41°–55°, based on the requirement in 24444:2019, in order to balance ITA° influences on results. The influence of ITA° values on LED‐HDRS results was known from results of P8, which were previously reported by Wiora et al. [13].

Application areas (test sites) on the back of the subjects were 50 × 80 mm large and were demarcated with a skin friendly marker prior to the DRS measurements. Prior to the sunscreen application, 10 DRS spectra were taken and averaged, matching the application areas, which served as a reference measurement $R_{0}$ on unprotected skin in Equation 1.

Sunscreen application was performed following the procedures described in ISO 24444, and sunscreen samples were randomized on the different skin areas. For reproducible positioning of the fibre probe within the application areas, a template fitting to the application areas with 12 holes was used, into which the fibre probes fitted. Each application area was measured at 10 positions within the template. The two remaining positions were used in case the measurements had to be deleted due to operating errors.

### In vitro measurements

In vitro measurements were performed according to ISO 24443 [15] procedure, using moulded HD6 PMMA plates (Helioscreen, Marseille, France) and Suntest CPS+ (Atlas, Ametek, Illinois, USA) devices for UV irradiation. Transmission measurements were performed with UV2000S devices (Labsphere, Inc., North Sutton, NH, USA) in two labs as well as two customized setups qualified for ISO 24443 [17, 18], as described in Table S1. In contrast to the ISO 24443 procedure, SPF values are not available for scaling the spectra in order to get UVA‐PF_0_ and therefore UVA‐PF_DRS_ (UVA‐PF calculated from DRS in vivo absorbance spectrum) is used to calculate the irradiation dose instead.

### HDRS principle

The general HDRS principle for LED‐HDRS is the same as described by ISO 23698 [11].

**FIGURE 1:**
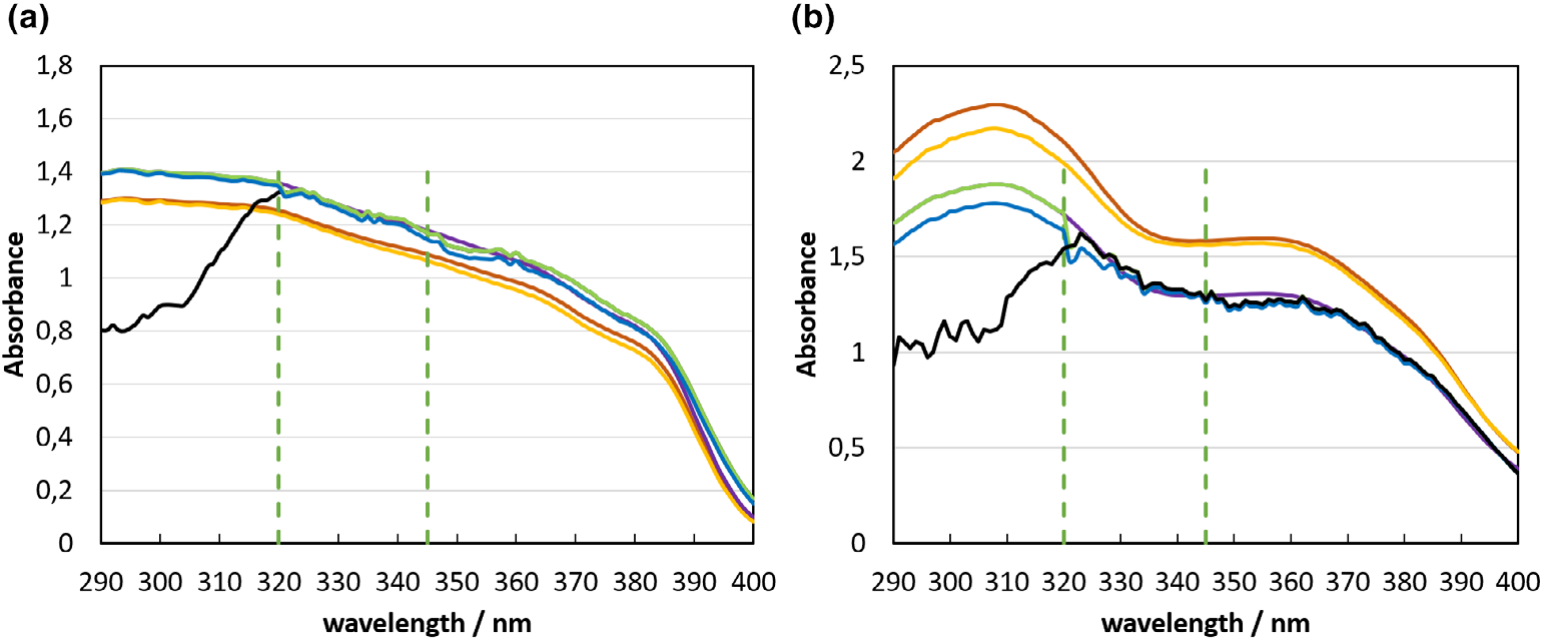
Example of spectral hybridization for individual volunteers with P5 (a) and P8 (b). $A_{\mathrm{DRS}}\left(\lambda \right)$ in vivo DRS spectrum (black line), $A_{\mathrm{vt}0}$(λ) pre‐exposure in vitro spectrum (red line), $A_{\mathrm{vt}1}$(λ) post‐exposure in vitro spectrum (orange line), $A_{\mathrm{vt}0}\cdot c_{\mathrm{Ai}}$ pre‐exposure in vitro spectrum scaled to $A_{\mathrm{DRS}}$(λ) (purple line). $A_{\text{HDRS}0}\left(\lambda \right)$ hybrid spectrum without photodegradation (green line), $A_{\text{HDRS}}\left(\lambda \right)$ hybrid spectrum with photodegradation correction (blue line), ${\mathrm{HW}}_{\min}$ and ${\mathrm{HW}}_{\max}$ minimum and maximum wavelength of hybridization range (vertical green dotted lines).

The in vitro spectra are scaled to match the absolute height of the in vivo DRS‐spectra in absorbance units. The scaling factor is determined by a ratio of both spectra in the hybridization range, and the in vitro spectrum replaces the insufficiently measured UVB part of the in vivo spectrum. In the ALT‐SPF, a hybridization range of 320–330 nm was used for LED‐HDRS. This was extended to 320–345 nm in the re‐evaluation study for improved stability of the hybridization, since the previous range often contained insufficient signal close to the short wavelength limit. The hybridization principle is shown with example spectra in Figure 1.

The resulting hybrid absorbance spectrum together with the calibration factors in Equation 4 is used to calculate SPF_HDRSi_ and UVA‐PF_HDRSi_ for each volunteer according to the equations in ISO 23698. For the exemplary P5 measurement, the SPF_HDRSi_ = 32 (with additional bias correction) is well within the acceptance range of 23.7 to 37.4, which is required for the panel average SPF_HDRS_. The exemplary P8 measurement for one volunteer with SPF_HDRSi_ = 49.6 (with additional bias correction) is also in the acceptance range of 43.9 to 82.3, which has to be met for the SPF_HDRS_ of the whole panel.

**FIGURE 2:**
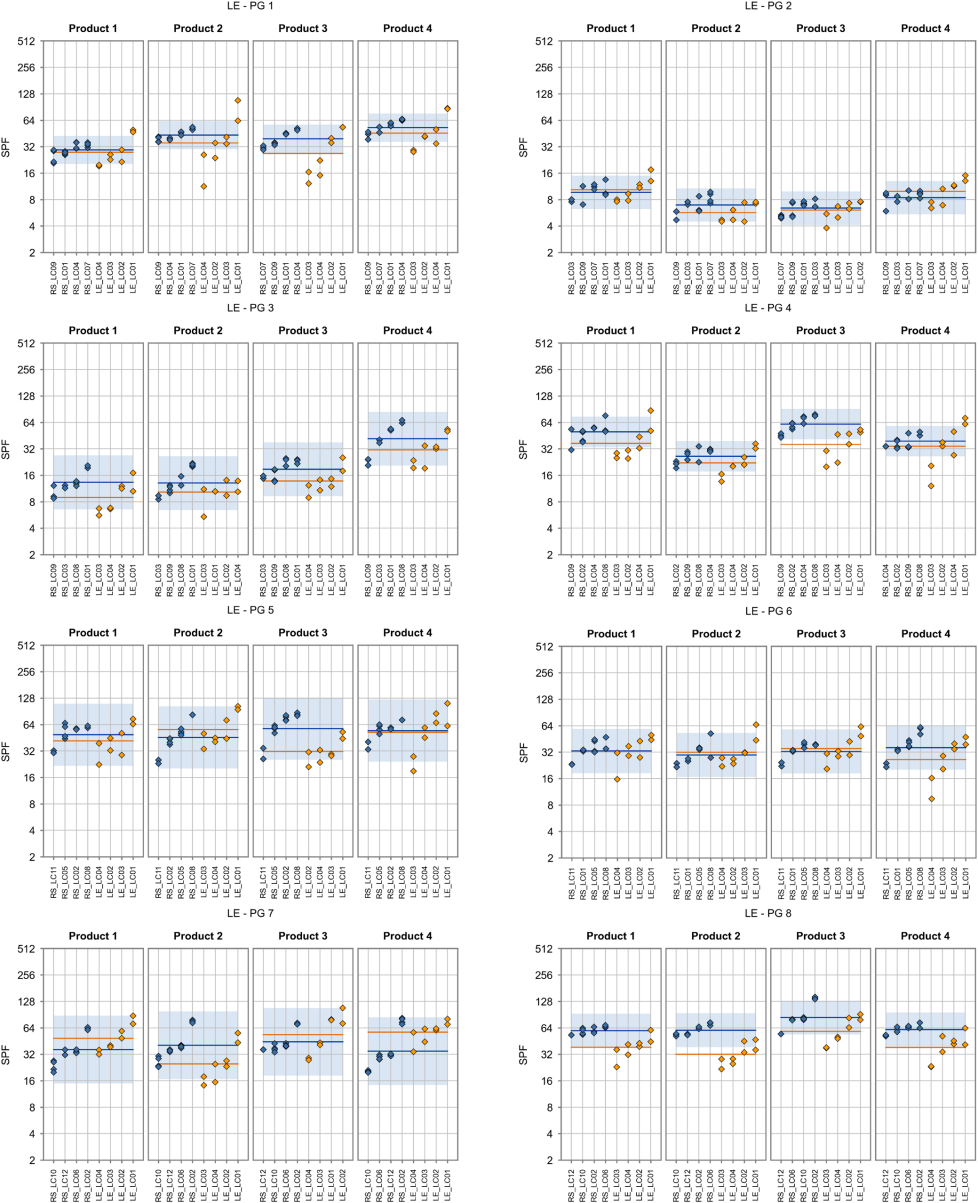
Individual ISO 24444 test results (base‐2 logarithmic scale) are displayed as blue diamonds, and the product‐specific ISO 24444 mean value is shown as a horizontal blue line. The prediction range for ISO 24444 (calculated from the reproducibility standard deviation) is displayed as a blue band. For the alternative method, individual test results and product‐specific mean values are displayed in orange. Lab codes are shown along the x‐axis. Test values are sorted in ascending lab‐mean order.

**FIGURE 3:**
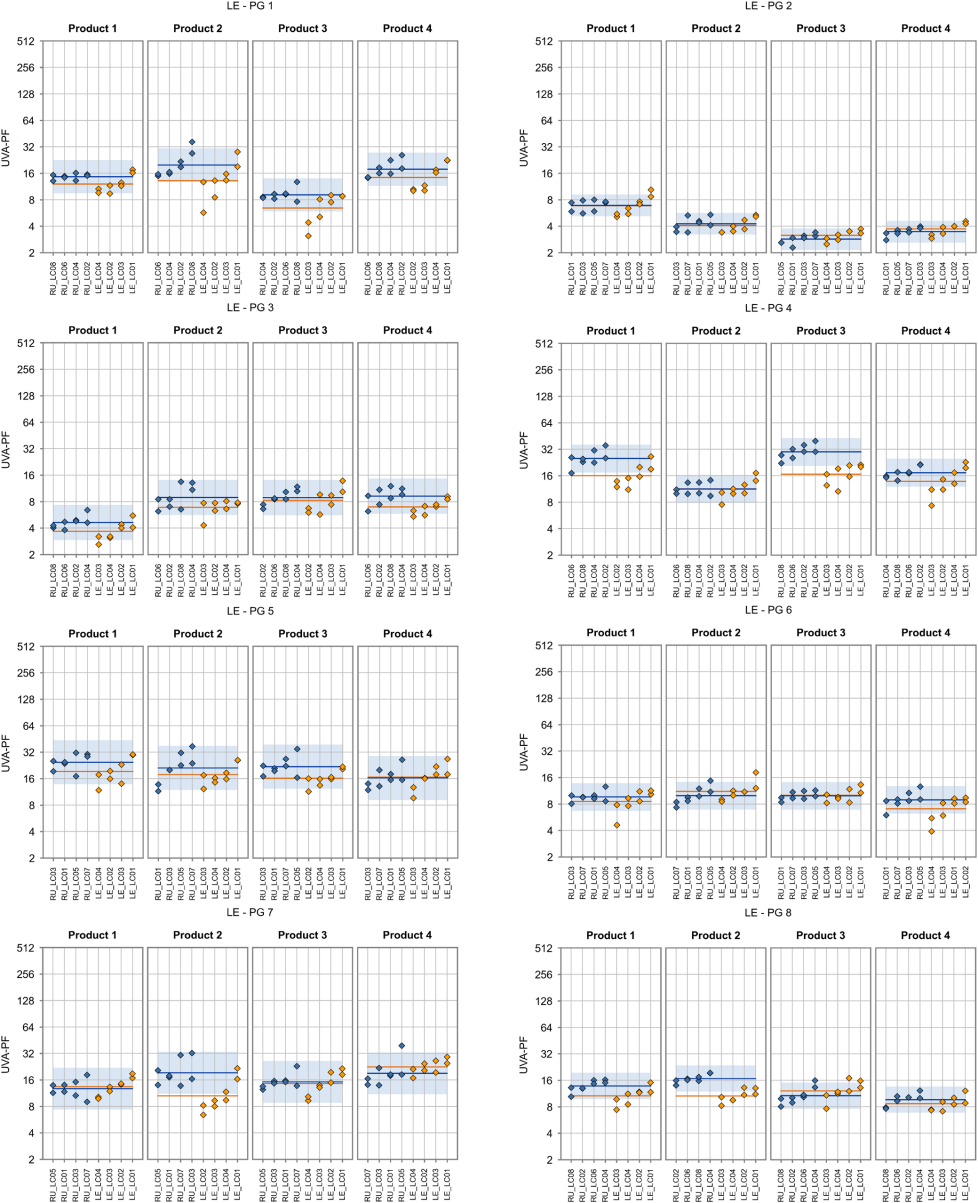
Individual ISO 24443 test results (base‐2 logarithmic scale) are displayed as blue diamonds, and the product‐specific ISO 24443 mean value is shown as a horizontal blue line. The prediction range for ISO 24443 (calculated from the reproducibility standard deviation) is displayed as a blue band. For the alternative method, individual test results and product‐specific mean values are displayed in orange. Lab codes are shown along the x‐axis. Test values are sorted in ascending lab‐mean order.

In order to account for photodegradation, a linear correction was applied to the ALT‐SPF study 1 data (Figures 2 and 3 and Tables 4, 5, 6, 7) and all previous LED‐HDRS studies by multiplying the transmission spectra by the factor spectral ratio of photodegradation (SRPD),

(5) $$\text{SRPD}=\frac{10^{A_{\mathrm{vt}0}}}{10^{A_{\mathrm{vt}1}}}$$

which is the same as the first of three equations in ISO 23698, corresponding to a ratio of transmissions. Here $A_{\mathrm{vt}0}$ and $A_{\mathrm{vt}1}$ are the in vitro absorbance spectra before and after irradiation. Equation 5 was applied to all samples equally.

**TABLE 4:** Assessment of original results of the LED‐HDRS method (alt) against ISO 24444:2019 for criteria 1 to 5.

(a)						
Product group	Criterion 1 [%]	Criterion 2 [%]	Criterion 3 [%]	Criterion 4 [%]	Criterion 5 [%]	All criteria
1	304.4	161.3	304.3	30.9	50.6	
2	119.6	75.7	125.1	1.9		
3	101.6	98.2	101.6	69.5		
4	223.5	134.7	223.5	51.9		
5	110.9	121.8	122.7	25.4		
6	143.5	121.9	143.5	7.8		
7	99.2	128.3	122.5	18.4		
8	141.4	86.7	141.4	127.7		

b)													
Product group	Criterion 1			Criterion 2			Criterion 3			Criterion 4			
	*S*_*R*_ alt	*S*_*R*_ ref	Validation	*S*_*L*,pers_ alt	*T*	Validation	Augm. *S*_*R*_ alt	Augm. *S*_*R*_ ref	Validation	AL	Bias alt	DL	Validation
1	0.56	0.18		0.48	0.3		0.56	0.18		0.00	−0.20	0.64	OK
2	0.26	0.22		0.23	0.3	OK	0.27	0.22		0.00	−0.01	0.33	OK
3	0.36	0.35		0.29	0.3	OK	0.36	0.35		0.00	−0.31	0.44	OK
4	0.45	0.20		0.40	0.3		0.45	0.20		0.00	−0.28	0.55	OK
5	0.45	0.41		0.37	0.3		0.50	0.41		0.00	−0.15	0.59	OK
6	0.42	0.29		0.37	0.3		0.42	0.29		0.00	−0.04	0.53	OK
7	0.44	0.44	OK	0.38	0.3		0.54	0.44		0.00	0.12	0.68	OK
8	0.31	0.22		0.26	0.3	OK	0.31	0.22		0.00	−0.47	0.37	

(c)			
Mean AL	Criterion 5		
	Variation of bias alt across product goups (SD)	CL	Validation
0.00	0.08	0.16	OK

*Note*: (a) Colour codes: Green = the criterion was met, Orange = the criterion was ‘almost met’ (ratio less than or equal to 110%), Red = the criterion was not met. (b) $s_{R}$ alt corresponds to the reproducibility standard deviation of the alternative method, $s_{R}$ ref corresponds to reproducibility standard deviation of the reference method $s_{L,\text{pers}}$ corresponds to the persistent lab bias, AL corresponds to the acceptability limit, DL is the decision limit, CL is the consistency limit.

**TABLE 5:** Precision estimates and product‐specific means SPF values for each product group.

Product group	Number of		Precision (ln SPF)								Product‐specific mean values (SPF)			
	Results	Labs	$s_{R}$	$s_{L}$	$s_{L,\mathrm{per}s}$	$s_{L,\text{produ}\mathrm{ct}}$	$s_{L,F2}$	$s_{L,F2}$	$s_{L,F3}$	$s_{r}$	*m*_1_	*m*_2_	*m*_3_	*m*_4_
1	32	4	0.56	0.44	0.48	0.27	0.12	0.09	0.14	0.08	27.6	35.2	26.9	45.6
2	32	4	0.26	0.23	0.23	0.05	0.00	0.00	0.00	0.12	10.3	5.7	6.1	9.9
3	32	4	0.36	0.22	0.29	0.18	0.10	0.15	0.09	0.09	9.0	10.3	13.8	31.2
4	32	4	0.45	0.37	0.40	0.13	0.00	0.11	0.12	0.14	37.0	22.2	36.0	34.3
5	32	4	0.45	0.29	0.37	0.25	0.13	0.18	0.00	0.09	41.6	56.0	31.6	52.2
6	32	4	0.42	0.34	0.37	0.14	0.00	0.08	0.11	0.14	33.0	32.0	35.0	26.3
7	32	4	0.44	0.35	0.38	0.21	0.10	0.08	0.10	0.05	48.7	24.9	53.6	57.2
8	32	4	0.31	0.25	0.26	0.13	0.00	0.00	0.08	0.12	38.5	32.0	58.3	38.3

*Note*: The precision parameters s, correspond to a standard deviation in units of $\ln\left(\mathrm{SPF}\right)$, $s_{R}$ = reproducibility, $s_{L}$ = lab bias, $s_{L,\text{pers}}$ = persistent lab bias, $s_{L,\text{Produc}t}$ = product related lab bias, $s_{L,\mathrm{Fi}}$ = factor related lab bias, $s_{r}$ = repeatability. The product‐specific mean values m_1_ to m_4_ correspond to the 4 products per product group.

**TABLE 6:** Assessment of original results of the LED‐HDRS method (alt) against ISO 24443: 2021 for criteria 1 to 5.

a)						
Product group	Criterion 1 [%]	Criterion 2 [%]	Criterion 3 [%]	Criterion 4 [%]	Criterion 5 [%]	All criteria
1	178.0	90.0	178.0	89.9	45.2	
2	133.4	55.9	133.4	12.6		
3	99.5	59.7	99.5	89.7		
4	157.7	85.3	199.0	103.3		
5	102.2	83.5	102.4	56.9		
6	136.8	72.6	146.3	20.4		
7	111.5	76.6	161.7	36.2		
8	116.5	62.0	173.6	71.3		

b)													
Product group	Criterion 1			Criterion 2			Criterion 3			Criterion 4			
	*S*_*R*_ alt	*S*_*R*_ ref	Validation	*S*_*L*,pers_ alt	*T*	Validation	Augm. *S*_*R*_ alt	Augm. *S*_*R*_ ref	Validation	AL	Bias alt	DL	Validation
1	0.39	0.22		0.27	0.3	OK	0.39	0.22		0.00	−0.29	0.32	OK
2	0.19	0.14		0.17	0.3	OK	0.19	0.14		0.00	−0.03	0.25	OK
3	0.23	0.23	OK	0.28	0.3	OK	0.23	0.23	OK	0.00	−0.21	0.24	OK
4	0.29	0.18		0.26	0.3	OK	0.36	0.18		0.00	−0.31	0.30	
5	0.30	0.29		0.25	0.3	OK	0.30	0.29		0.00	−0.18	0.31	OK
6	0.25	0.18		0.22	0.3	OK	0.26	0.18		0.00	−0.06	0.28	OK
7	0.30	0.27		0.23	0.3	OK	0.44	0.27		0.00	−0.11	0.30	OK
8	0.20	0.17		0.19	0.3	OK	0.30	0.17		0.00	−0.17	0.24	OK

c)			
Mean AL	Criterion 5		
	Variation of bias alt across product goups (SD)	CL	Validation
0.000	0.042	0.093	OK

*Note*: (a) Colour codes: Green = the criterion was met, Orange = the criterion was ‘almost met’ (ratio less than or equal to 110%), Red = the criterion was not met. (b) $s_{R}$ alt corresponds to the reproducibility standard deviation of the alternative method, $s_{R}$ ref corresponds to reproducibility standard deviation of the reference method $s_{L,\text{pers}}$ corresponds to the persistent lab bias, AL corresponds to the acceptability limit, DL is the decision limit, CL is the consistency limit.

**TABLE 7:** Precision estimates and product‐specific means UVA‐PF values for each product group.

Product group	Number of		Precision [ln UVA‐PF]								Product‐specific mean values [UVA‐PF]			
	Results	Labs	$s_{R}$	$s_{L}$	$s_{L,\mathrm{per}s}$	$s_{L,\text{produ}\mathrm{ct}}$	$s_{L,F1}$	$s_{L,F2}$	$s_{L,F3}$	$s_{r}$	*m*_*1*_	*m*_*2*_	*m*_*3*_	*m*_*4*_
1	32	4	0.39	0.21	0.27	0.26	0.12	0.05	0.12	0.09	12.1	13.2	6.4	14.4
2	32	4	0.19	0.17	0.17	0.07	0.02	0.00	0.02	0.06	6.8	4.1	3.2	3.7
3	32	4	0.23	0.12	0.18	0.11	0.02	0.11	0.06	0.09	3.7	6.9	8.3	6.9
4	32	4	0.29	0.21	0.26	0.09	0.00	0.13	0.07	0.10	16.0	11.3	16.6	13.8
5	32	4	0.30	0.19	0.25	0.15	0.08	0.14	0.04	0.05	19.2	17.8	16.1	16.6
6	32	4	0.25	0.19	0.22	0.06	0.00	0.07	0.08	0.10	8.5	11.1	10.0	7.1
7	32	4	0.30	0.20	0.23	0.19	0.03	0.07	0.08	0.05	13.4	10.5	14.5	22.5
8	32	4	0.20	0.14	0.19	0.00	0.05	0.08	0.06	0.07	10.6	10.6	12.1	8.7

*Note*: The precision parameters s, correspond to a standard deviation in units of $\ln\left(\mathrm{UVA}-\mathrm{PF}\right)$, $s_{R}$ = reproducibility, $s_{L}$ = lab bias, $s_{L,\text{pers}}$ = persistent lab bias, $s_{L,\text{Product}}$ = product related lab bias, $s_{L,\mathrm{Fi}}$ = factor related lab bias, $s_{r}$ = repeatability. The product‐specific mean values m_1_ to m_4_ correspond to the 4 products per product group.

In the ISO 23698, different equations for SRPD are used, depending on the grade of photoinstability described by the value of SRPD in the UVA and UVB, which are then multiplied by the absorbance spectrum.

For comparison, the results of the ALT‐SPF study 1 (Figures 2 and 3, Table 4, 5, 6) were recalculated by using the case‐dependent three equations of ISO 23698 and the larger hybridization range. The results are presented in Tables 8 and 9.

For the re‐evaluation study (Tables 10 and 11), the results were submitted with the case‐dependent equations of ISO 23698.

*C*_LED_ was set to 0.8695 for the linear correction of photodegradation based on results of previous studies [12, 13].

In order to account for the higher correction effect of the case‐dependent non‐linear equations in ISO 23698, *C*_LED_ was set to 0.9174 to achieve the same average of values as with the original calibration. Before the re‐evaluation study, the negative average bias of the data was not corrected due to unknown effects of the training and experimental improvements. Since the re‐evaluation study confirmed the systematic negative bias, the overall bias was corrected retrospectively by the statistician (QuoData, Dresden, Germany) based on the ALT‐SPF data set and calculated via the ‘MU‐Hampel’ [19], a robust estimator which also takes into consideration the standard errors. For SPF, the overall bias was calculated as −0.171 ln SPF. For UVA‐PF, the overall bias was calculated as −0.162 ln UVA‐PF. Given the degree of agreement between the two overall bias values and the fact that the difference was not statistically significant, it was decided to apply one common correction term for both SPF and UVA‐PF (−0.164 ln SPF or UVAPF, respectively). It leads to an additional factor *e* ^0.164^ for the bias optimized *C*_cal_ in Equation 4.

## RESULTS AND DISCUSSION

### Results of LED‐HDRS versus ISO 24444 (ALT‐SPF study 1, first results without improvements)

This section describes the results from study 1 as received after the first evaluation without any improvements that were performed retrospectively on the data. The improvements are presented in Table 8. The results of SPF_HDRS_ for each lab and setting compared with the gold standard method ISO 24444 are shown in Figure 2 for each product group separately.

With the exception of the mineral group (prior to the bias correction shown in Table 8), the results show a small bias and small variation of bias across product groups, if results of all labs are averaged. However, there are considerable lab‐specific discrepancies, especially for product groups 1 and 4.

**TABLE 8:** Assessment of recalculated SPF results of the LED‐HDRS method against ISO 24444 for criteria 1–5.

Data set	Product group	Criterion 1 [%]	Criterion 2 [%]	Criterion 3 [%]	Criterion 4 [%]	Criterion 5 [%]	All criteria
ALT‐SPF (study 1 with bias corr) hybridization range: 320–330 nm	1	304.4	161.3	304.3	5.4	50.6	
	2	119.6	75.7	125.1	47.6		
	3	101.6	98.2	101.6	32.4		
	4	223.5	134.7	223.5	21.8		
	5	110.9	121.8	122.7	2.3		
	6	143.5	121.9	143.5	23.1		
	7	99.2	128.3	122.5	42.7		
	8	141.4	86.7	141.4	83.4		
SPF 1 hybridization range: 320–345 nm	1	272.6	147.2	274.5	34.4	33.6	
	2	105.6	66.9	111.8	45.6		
	3	101.5	92.3	101.5	45.0		
	4	213.9	129.2	213.9	29.1		
	5	119.1	111.9	121.9	18.3		
	6	136.8	112.8	140.5	8.2		
	7	82.4	108.0	102.9	23.9		
	8	159.9	102.8	159.9	69.1		
SPF 2 hybridization range: 320–345 nm photodegradation as in ISO 23698 and new calibration	1	268.5	136.9	268.5	42.9	29.9	
	2	119.2	75.9	124.0	50.4		
	3	117.7	106.2	117.7	44.0		
	4	178.9	99.7	198.0	60.0		
	5	119.4	128.3	123.1	11.5		
	6	151.2	121.0	166.3	2.9		
	7	88.1	105.0	115.7	30.4		
	8	201.0	131.3	201.0	41.2		

*Note*: Colour codes are as in Table 4. For all results in this table, a correction for systematic bias of −ln 0.164 ln SPF was performed by QuoData.

Tables 4 and 5 provide the results of the detailed assessment of the LED‐HDRS method against ISO 24444. For a given criterion and product group, the percentage value provided in Table 4a was calculated as the ratio of the relevant statistical parameter for the alternative method and the threshold in Table 4b,c.

The reproducibility standard deviation of the alternative method $s_{R}$ alt (total random variation) is equal to the reference method's for product group 7 (see criterion 1). For the remaining seven product groups, criterion 1 is not met. In particular, $s_{R}$ alt is very large for product group 1 (0.56).

The persistent lab bias $s_{L,\text{pers}}$ (i.e. product‐specific effects on lab bias are discarded) is less than the threshold for PG 2, 3, and 8 (see criterion 2). The augmented reproducibility standard deviation of the alternative method (i.e. taking into account the variation of bias from product to product) is higher than $s_{R}$ alt for 8 product groups (see criterion 3). Criterion 4 is met for seven product groups, where the bias ranges between −0.31 and 0.12. For product group 8, the bias is larger than the decision limit DL. Overall, it can be stated that the overall bias tends to be slightly negative (see criteria 4 and 5). Product groups 3 (nearly) meets all criteria.

### Results of LED‐HDRS versus ISO 24443 results (ALT‐SPF Study 1, first results without improvements)

This chapter describes the results of study 1 as received after the first evaluation without any improvements that were performed retrospectively on the data. The improvements are presented in Table 9.

**TABLE 9:** Assessment of recalculated UVA‐PF results of the LED‐HDRS method against ISO 24443 for criteria 1–5.

Data set	Product group	Criterion 1 [%]	Criterion 2 [%]	Criterion 3 [%]	Criterion 4 [%]	Criterion 5 [%]	All criteria
ALT‐SPF (study 1 with bias corr) hybridization range: 320–330 nm	1	178.0	90.0	178.0	39.0	45.2	
	2	133.4	55.9	133.4	79.4		
	3	99.5	59.7	99.5	20.0		
	4	157.7	85.3	199.0	49.0		
	5	102.2	83.5	102.4	3.5		
	6	136.8	72.6	146.3	39.0		
	7	111.5	76.6	161.7	19.3		
	8	116.5	62.0	173.6	3.7		
UVA‐PF 1 hybridization range: 320–345 nm	1	172.1	101.5	176.4	61.2	62.4	
	2	128.4	55.0	128.4	67.1		
	3	102.8	61.8	102.8	31.1		
	4	159.7	86.7	198.2	62.6		
	5	108.6	81.6	108.6	25.5		
	6	151.6	77.5	151.6	19.9		
	7	99.7	66.5	149.3	4.5		
	8	137.0	71.9	176.9	27.5		
UVA‐PF 2 hybridization range: 320–345 nm photodegradation as in ISO 23698 and new calibration	1	172.9	85.8	172.9	85.3	96.6	
	2	138.8	58.7	138.8	76.7		
	3	106.8	61.4	106.8	37.6		
	4	155.5	73.9	211.5	91.0		
	5	104.9	86.0	106.5	8.3		
	6	180.8	79.0	187.9	10.5		
	7	90.7	55.9	155.9	26.9		
	8	149.9	76.2	178.1	3.2		

*Note*: Colour codes are as in Table 4. For all results in this table, a correction for systematic bias of −ln 0.164 to ln UVA‐PF was performed by QuoData.

The results of UVA‐PF_HDRS_ for each lab and setting compared with the reference in vitro method ISO 24443 are shown in Figure 3 for each product group separately. In summary, the results show a small bias and small variation of bias across product groups if results of all labs were averaged. Lab LC01 performs considerably better than the other laboratories, which display a clear tendency towards a negative bias.

Table 6 provides the detailed results of the assessment of the LED‐HDRS method against ISO 24443. For a given criterion and product group, the percentage value provided in Table 6a was calculated as the ratio of the relevant statistical parameter for the alternative method and the threshold, which is described in Table 6b,c.

The reproducibility standard deviation of the alternative method $s_{R}$ alt (total random variation) is lower than the reference method's for product group 3 (see criterion 1). For product group 5, $s_{R}$ alt is only slightly higher than $s_{R}$ ref., and is not considered critical. For the remaining six product groups, criterion 1 is clearly not met. In particular, $s_{R}$ alt is very large for product group 1 (= 0.39).

The persistent lab bias $s_{L,\text{pers}}$ (i.e. product‐specific effects on lab bias are discarded) is no higher than 0.26 (and thus lower than the threshold of 0.3) (see criterion 2). However, it is the dominant component of reproducibility standard deviation of the alternative method $s_{R}$ alt for all product groups. This indicates that lab bias must be reduced even though criterion 2 is met. The augmented reproducibility standard deviation of the alternative method (i.e. taking into account the variation of bias from product to product) is equal to or only slightly larger than $s_{R}$ alt for 6 product groups (see criterion 3), indicating that bias within a product group tends to be consistent across products. Criterion 3 is met for product group 3. Criterion 4 is met for seven product groups, where the bias ranges between −0.29 and 0.03. For product group 4, the bias of 0.31 is slightly larger than the decision limit of 0.3. Overall, it can be stated that the bias tends to be consistent (negative) across product groups. This consistency is reflected in the standard deviation of the bias, which is 0.042 and less than half of the consistency limit CL of 0.093. Product groups 3 and 5 (nearly) meet all criteria.

### Improved results of LED‐HDRS versus ISO 24444 and ISO 24443 (ALT‐SPF study 1 data recalculated)

#### Improving on hybridization range

The rationale behind the choice of the hybridization range is that hybridization as close to the UVB as possible allows using as much of the in vivo spectrum as possible. At the same time, the signal is highly dependent on the sunscreen and the skin pigmentation. Our approach was therefore to fix the hybridization range at the wavelengths with maximal irradiation intensity (320–345 nm). The hybridization procedure is as described in ISO 23698; however, the two HDRS methods described there use different hybridization ranges. Monochromatic HDRS uses a dynamic signal‐dependent hybridization range of 5 nm in the range of 322 to 350 nm, and polychromatic HDRS uses only a single wavelength (345 nm) to determine the scaling factor.

Table 8 shows the effect of broadening the hybridization range on the SPF_HDRS_, and Table 9 shows the effect on the UVA‐PF_HDRS_ correspondingly.

For all results in this table, a correction for systematic bias of −ln 0.164 to ln UVA‐PF was performed by QuoData, which has a minor impact on criterion 4 and does not change other criteria. ALT‐SPF are the originally submitted data with a hybridization range of 320 to 330 nm. UVA‐PF 1 is calculated with the same calibration as the original results, but with a larger hybridization range (320–345 nm).

The effect of broadening the hybridization range improves the overall results of SPF_HDRS_ slightly and PGs 3 and 7 almost met the criteria (corresponding to orange colour code), see SPF 1 in Table 8.

#### Comparison of different corrections for photodegradation

In the original results of the ALT‐SPF study, the correction for photodegradation was performed linearly by using the first equation in ISO 23698 (for SRPD > 0.8, see Equation 5) for all samples. However, as Equation 5 corresponds to a ratio of transmissions, the factor was multiplied with the spectra in transmission units and not with absorbance spectra as in ISO 23698. Table 8 shows the effect of the change of photodegradation equations on the SPF_HDRS_, and Table 9 shows the effect on the UVA‐PF_HDRS_ correspondingly. In contrast to SPF 1 and UVA‐PF 1, the results SPF 2 and UVA‐PF 2 are obtained using the case‐dependent photodegradation exactly as in ISO 23698. For the SPF 2 results, the results get slightly worse as compared to SPF 1. For the UVA‐PF 2 results given in Table 9, the changes as compared to UVA‐PF 1 are negligible.

**FIGURE 4:**
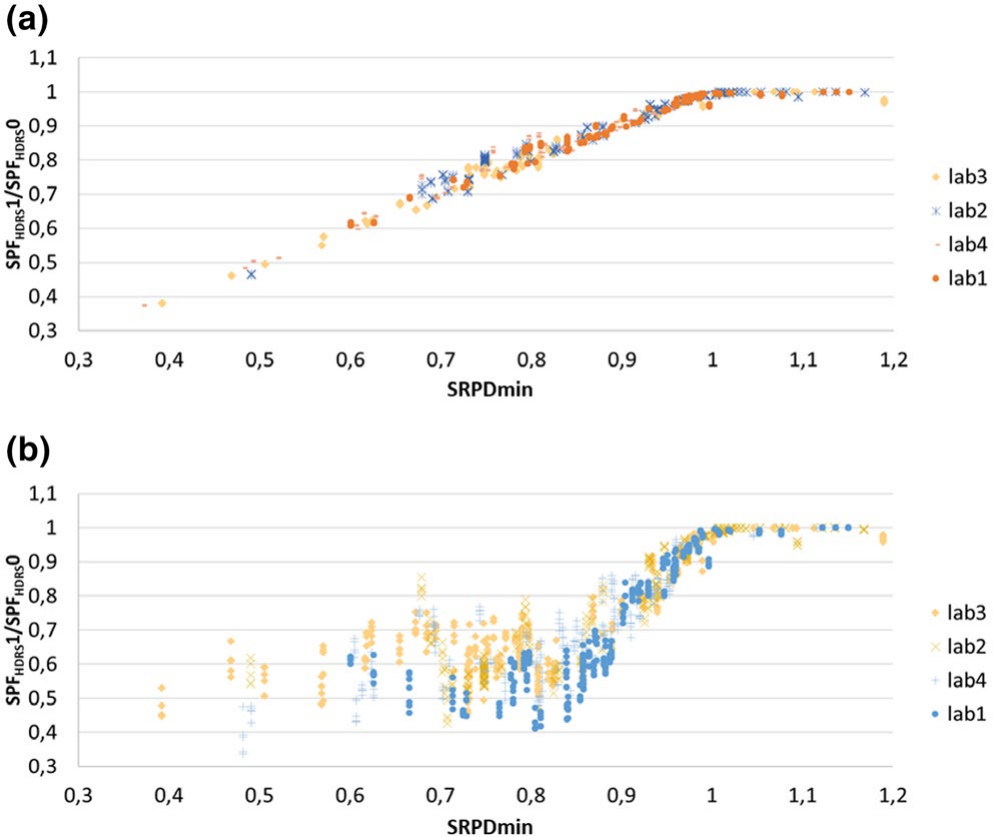
Correction effect as SPF_HDRS1_/SPF_HDRS0_ shown for the ALT‐SPF data of the LED‐HDRS labs. (a) Correction of T with Equation 5. for all samples (b) Correction of A dependent on SRPD as described in ISO 23698.

The effect of the different corrections for photodegradation can be better visualized by Figure 4, where it is shown that the linear correction in T leads to a lower interlaboratory variance of correction effects. One hypothesis may be that for our data, small differences in the in vitro absorbance spectra lead to relatively large differences in correction effect. An important difference of both results in Figure 4 is that the equations in ISO 23698 applied to our data implicate a different photodegradation under in vitro conditions than during DRS measurement conditions. Those samples that are relatively stable under in vitro conditions (corresponding to SRPD > 0.8) experience the strongest correction effect down to approximately 60% of the uncorrected value in the HDRS in vivo measurement. However, a higher photodegradation under in vitro conditions (corresponding to SRPD <0.8) seems not to reduce the corrected HDRS results further than those with SRPD = 0.8.

### Device comparison

**FIGURE 5:**
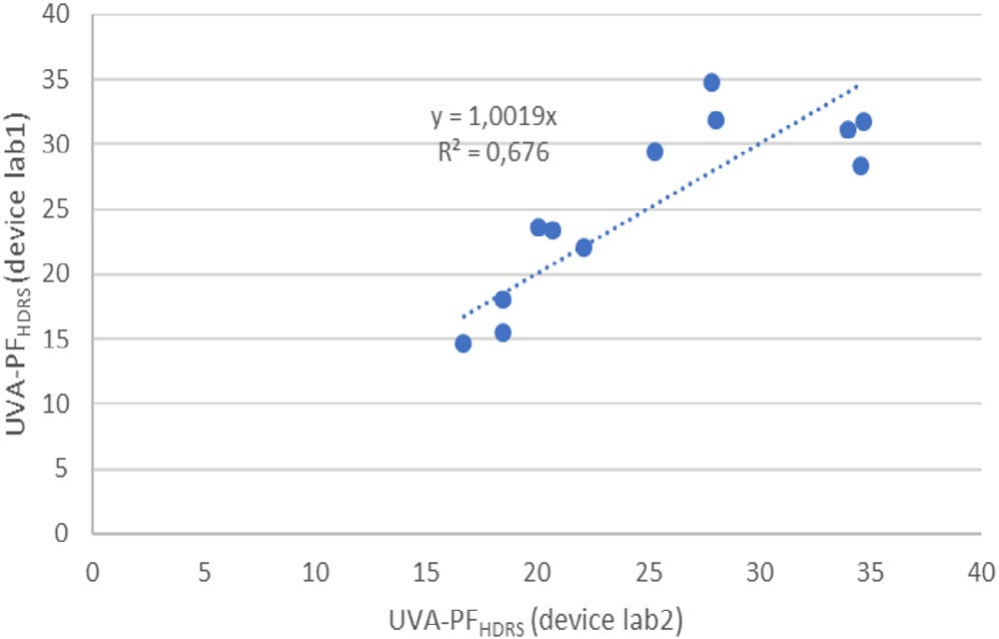
Correlation of UVA‐PF_HDRS_ values measured with two devices in one lab, with the same operators and within the same measurement areas with two samples, three positions for each sample and volunteer (*n* = 2).

In order to investigate the possible influences of potential technical differences, two of the four devices from the ALT‐study were operated simultaneously in one lab on the same volunteer, within the same measurement area with the same in vivo operator and using the same in vitro spectra. With both devices being in two separate labs during the ALT‐SPF study 1, the average bias of SPF_HDRS_ values from reference SPF values was as large as −9% (lab 2) versus +32% (lab 1). For the device comparison, P8 and one sample from the ALT‐SPF study 1 (sample no. 5–2) were measured, using each device to measure 6 out of the 12 available measurement positions in each measurement area, in order to exclude the influence of product application and skin site. Two volunteers were measured with 3 repetitions and two sunscreens each. The calculated linear regression (slope of 1.002, *R* ^2^ = 0.67) and correlation (*r* = 0.84, *p* < 0.001) indicate good evidence that both devices give the same results, see Figure 5. Consequently, the differences between these two labs observed in the first round of the ALT‐SPF study could be linked to differences either in handling by the operators, volunteers' skin influences, as well as to differences in in vitro spectra. From other experimental results, we know that all three factors are relevant for interlaboratory variance.

**FIGURE 6:**
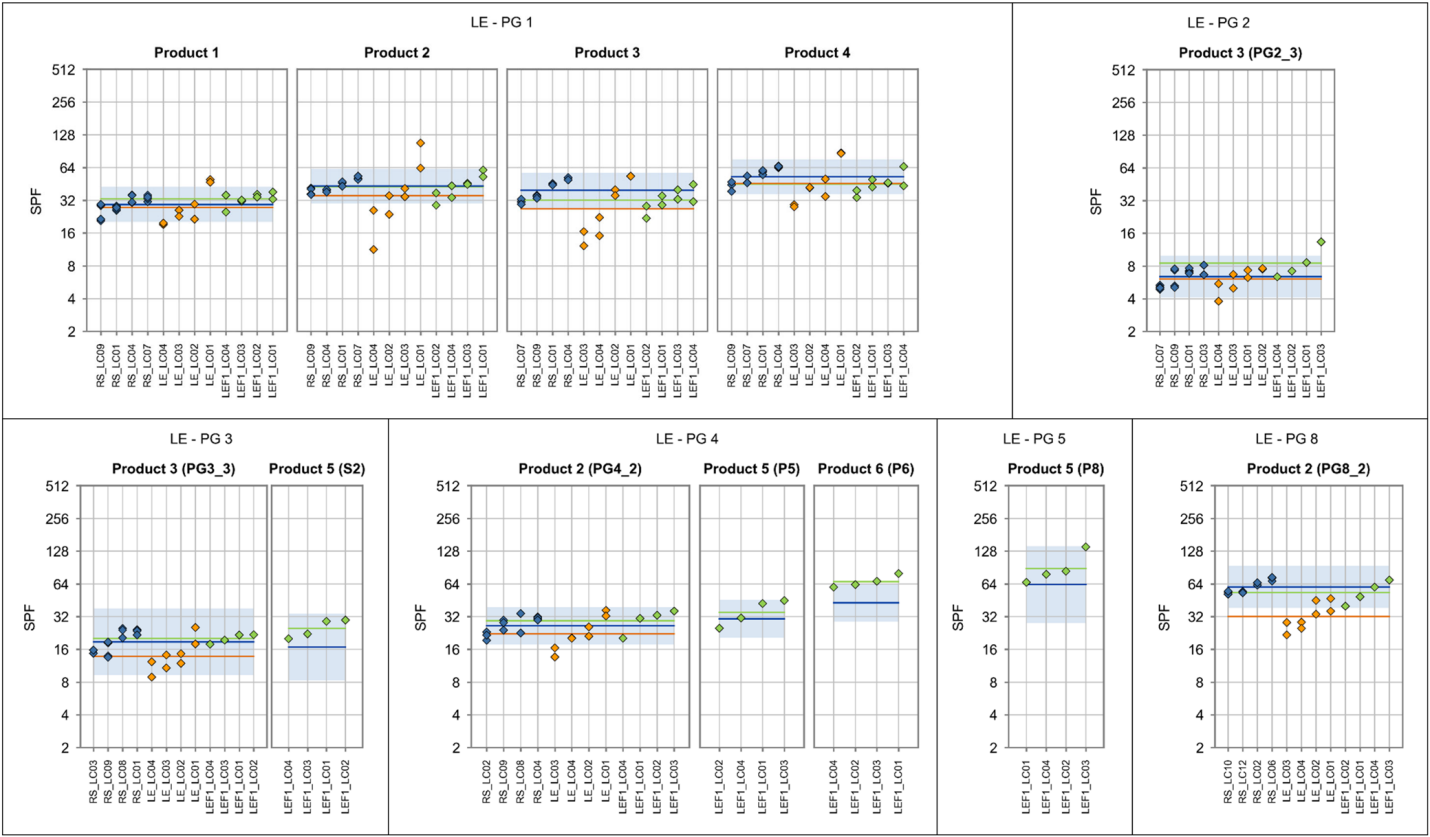
Individual ISO 24444 test results (base‐2 logarithmic scale) are displayed as blue diamonds, and the product‐specific ISO 24444 mean value is shown as a horizontal blue line. The prediction range for ISO 24444 (calculated from the reproducibility standard deviation) is displayed as a blue band. For the LED‐HDRS method, individual test results and product‐specific mean values are displayed in orange for ALT‐SPF study 1 and in green for re‐evaluation study 2 (with bias correction). Lab codes are shown along the *x*‐axis. Test values are sorted in ascending lab‐mean order.

**FIGURE 7:**
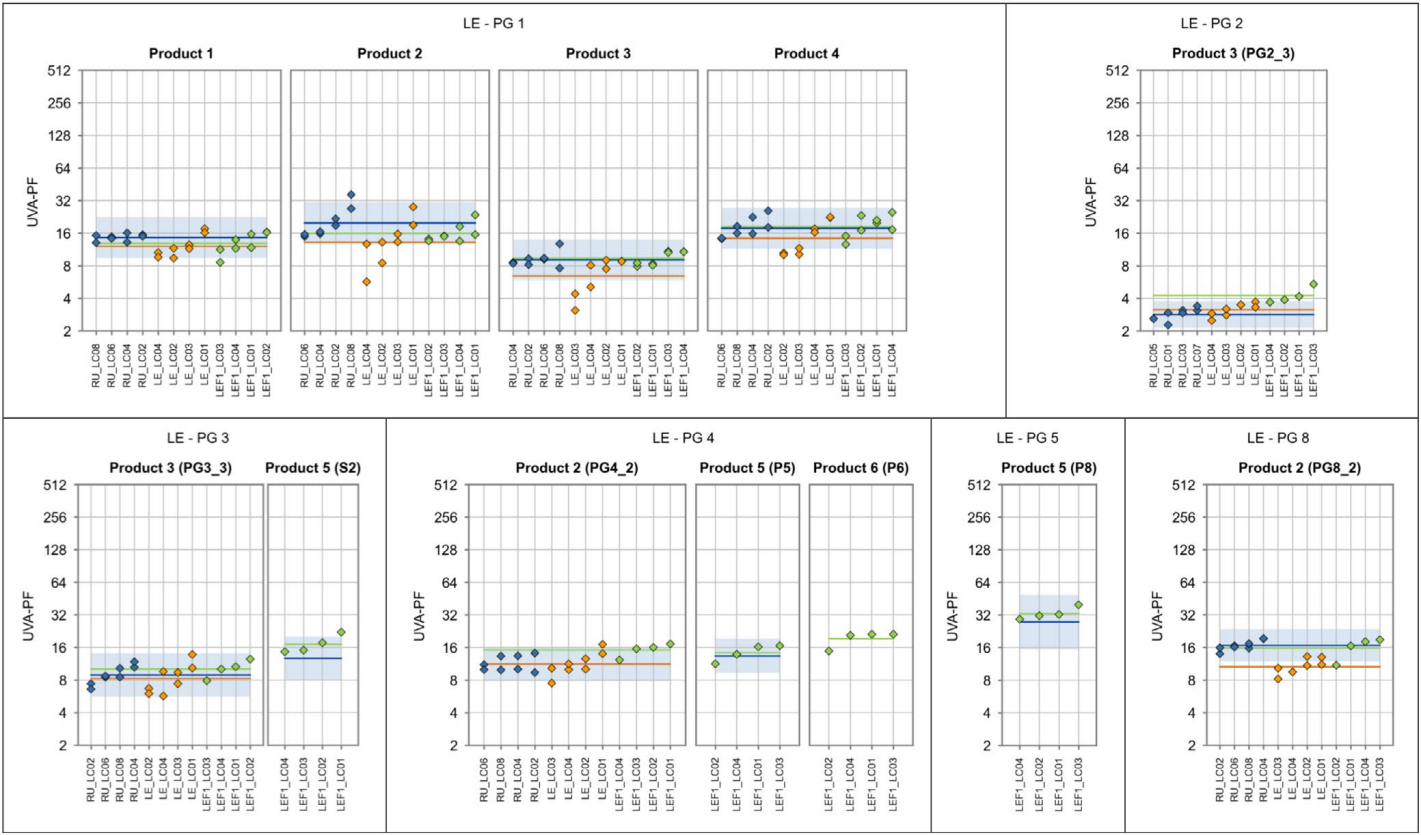
Individual ISO 24443 test results (base‐2 logarithmic scale) are displayed as blue diamonds, and the product‐specific ISO 24443 mean value is shown as a horizontal blue line. The prediction range for ISO 24443 (calculated from the reproducibility standard deviation) is displayed as a blue band. For the alternative method, individual test results and product‐specific mean values are displayed in orange for ALT‐SPF study 1 and in green for re‐evaluation study 2 (with bias correction). Lab codes are shown along the *x*‐axis. Test values are sorted in ascending lab‐mean order. The bias correction based on study 1 was applied to this graph.

The same experimental design was used in order to prove a device‐dependent bias for another lab (lab 4 in ALT‐SPF study), which could be corrected prior to submission of the data of the re‐evaluation study (but remains uncorrected in all results except in Figures 6 and 7 and Table 13).

### Results of LED‐HDRS versus ISO 24444 and ISO 24443 (Re‐evaluation study 2)

In order to confirm the improvements and changes in the method after the ALT‐SPF measurements, a re‐evaluation study was carried out with 16 blinded samples, including both ALT‐SPF samples and standard sunscreens. For 8 of these samples (from PG 1), the factorial study design was applied. The performance (after correction of systematic bias on the basis of the ALT‐SPF study 1 data) described by the criteria is shown in Tables 10 and 11 for PG 1. The results in Table 10 for SPF_HDRS_ show that criterion 1 is, with 111%, very close to meeting the acceptance criteria, criterion 2 is met, criterion 3 is close with 129% (this criterion was the most difficult for all methods) and criterion 4 is met.

**TABLE 10:** Product group 1—overview of performance assessment for SPF_HDRS_ after bias correction.

Study	Criterion 1 [%]	Criterion 2 [%]	Criterion 3 [%]	Criterion 4 [%]	All criteria
ALT‐SPF (study 1)	304.3	161.3	304.3	30.9	
Re‐evaluation (study 2), *n* = 10 with bias correction	111.2	38.6	128.8	140.1 (no bias corr) 40.1 (with bias corr)	

*Note*: Colour codes are as in Table 4.

**TABLE 11:** Product group 1—overview of performance assessment for UVA‐PF_HDRS_ after bias correction. Colour codes are as in Table 4.

Study	Criterion 1 [%]	Criterion 2 [%]	Criterion 3 [%]	Criterion 4 [%]	All criteria
ALT‐SPF (study 1)	178.0	90.0	178.0	89.9	
Re‐evaluation (study 2), *n* = 10 with bias correction	95.7	11.4	108.0	168.4 (no bias corr) 50.4 (with bias corr)	

*Note*: Colour codes are as in Table 4.

This means the reproducibility improved by a factor of about three and interlaboratory variability improved by a factor of four. Colour codes are as in Table 4.

The results in Table 11 for UVA‐PF_HDRS_ show that the criteria for a new alternative method are met, with the exception of criterion 3, which almost meets the acceptance criteria at 108%.

Figures 6 and 7 show the detailed results per sample and lab for SPF and UVA‐PF, respectively. In summary, most results improved considerably and most results are within acceptance limits. For the SPF results of study 2 (green), there is only one out of four labs outside the blue acceptance range for products 1–2 (one of two operators), 1–3 (one of two operators), 1–4 (one of two operators), 2–3, P5 (borderline), P8 (borderline) and P6 with two labs outside and one borderline. In the case of UVA‐PF values of study 2 (green), there is only one out of four labs that is outside the blue acceptance range for products 1–1 (one of two operators), S2, 4–2 (plus one lab borderline), 8–2, but three labs are too high for product 2–3 with the lowest UVA‐PF.

After an initial evaluation by QuoData, outliers were checked for possible errors in raw data.

We noted that a larger difference in the in vitro spectra of product 1–2 (represented by 2 blinded samples) measured in lab 1 caused the significant spread of UVA‐PF values, despite similar UVA_DRS_ values. This improved only slightly after a repeating the in vitro measurement after adding more volunteers for UVA‐PF_DRS_ (in the graph and results calculations, the in vivo spectra of the first *n* = 10 volunteers were used for consistency). For the value of S2 measured by lab 3, we noticed a copy/paste error in the in vitro measurement (pre‐ and post‐ irradiation were interchanged), which caused an improvement in a subsequent correction.

It shall be noted that the number of volunteers was limited to *n* = 10 due to the large number of samples for the labs. Consequently, the statistical criterion of ISO 23698 was not fulfilled for all samples. However, the samples with *c* > 17% for UVA‐PF_DRS_ were not the outliers outside the acceptance range in Figures 6 and 7, indicating that the lack of reproducibility was not the origin of these outliers. Still, in order to improve on remaining sources of interlaboratory variation, more volunteers could be added in the future, considering that ISO 23698 allows up to 15 volunteers.

A further option for future improvement of the bias of all product groups, described in Tables 12 and 13, is to determine the bias correction based on all data of study 1 and study 2 together.

**TABLE 12:** Product specific bias of SPF_HDRS_ against ISO 24444 after bias correction and standard deviation of ln [SPF].

Product group	Product	Product code	Mean SPF [ISO 24444]	Mean SPF_HDRS_ [Re‐evaluation]	Bias after correction [ln SPF_HDRS_]	SD [ln SPF_HDRS_]
1	1	PG1_1	29.3	33.0	0.12	0.13
	2	PG1_2	43.3	42.4	−0.02	0.24
	3	PG1_3	39.4	32.2	−0.20	0.22
	4	PG1_4	52.7	45.1	−0.16	0.19
2	3	PG2_3	6.4	8.5	0.29	0.32
3	3	PG3_3	18.7	20.1	0.07	0.10
	5	S2	16.8	24.8	0.39	0.20
4	2	PG4_2	26.3	29.3	0.11	0.25
	5	P5	30.6	34.9	0.13	0.28
	6	P6	43.0	67.1	0.45	0.13
5	5	P8	63.1	88.2	0.34	0.32
8	2	PG8_2	60.1	53.3	−0.12	0.24

**TABLE 13:** Product specific bias of UVA‐PF_HDRS_ against ISO 24443 after bias correction and standard deviation of ln [UV‐PF].

Product group	Product	Product code	Mean UVA‐PF [ISO 24443]	Mean UVA‐PF_HDRS_ [Re‐evaluation]	Bias after correction [ln UVA‐PF_HDRS_]	SD [ln UVA‐PF_HDRS_]
1	1	PG1_1	14.6	12.9	−0.12	0.23
	2	PG1_2	19.9	15.8	−0.23	0.19
	3	PG1_3	9.1	9.4	0.03	0.15
	4	PG1_4	17.8	18.5	0.04	0.23
2	3	PG2_3	2.9	4.2	0.40	0.17
3	3	PG3_3	8.9	10.1	0.13	0.19
	5	S2	12.7	17.1	0.30	0.19
4	2	PG4_2	11.3	15.2	0.29	0.15
	5	P5	13.4	14.3	0.07	0.18
	6	P6	–	19.3	–	0.17
5	5	P8	27.5	33.0	0.18	0.13
8	2	PG8_2	16.7	15.8	−0.06	0.25

## SUMMARY AND OUTLOOK

The results of the ALT‐SPF and further reported studies show that the HDRS principle, realised with a third technical approach (in addition to the two approaches described in ISO23698), namely polychromatic LED‐based illumination and a spectrally resolved detection with an imaging detector, is appropriate and leads to reasonable results.

The quantitative evaluation criteria 1 (reproducibility) and 3 (reproducibility and bias across product groups) were not yet met in the re‐evaluation study for the SPF but were very close to being met. For the UVA‐PF, all criteria were met except for bias 3, which was almost met. A large improvement was achieved from ALT‐SPF (study 1) to re‐evaluation (study 2). Further improvements by training with standard samples are important.

It should be noted that the number of volunteers was limited to *n* = 5 (ALT‐SPF study 1) and to *n* = 10 (re‐evaluation study 2) due to the large number of samples for the labs. Consequently, the statistical criterion of ISO 23698 was not fulfilled for all samples in each lab. Prior to the beginning of the ALT‐SPF study, a statistical test was carried out with old data in order to justify the use of *n* = 5 volunteers only, based on the availability of data from four independent labs and eight independent settings. Nevertheless, more volunteers would most likely improve interlaboratory variation and reproducibility in the future.

More UVA power can be implemented as new LEDs become available. Thereby, adaptation to requirements of UVA‐PFs higher than measured in the ALT‐SPF study seems an option for the future. If the maximum reflectance is still in the hybridization range of 320–345 nm, the UVA‐PF_HDRS_ could additionally be derived using the scaled in vitro spectrum in the whole UV range, instead of using the UVB part of the in vitro spectrum only as it was the case for the result in this manuscript.

It is recommended as a task for the future to further investigate the influence of the spectrum used in the in vitro measurement step to challenge the sunscreen products' photo degradation. The LED‐HDRS method and other earlier HDRS measurements [9] used the spectrum as described by ISO 24443:2021. This spectrum has a UVA to UVB ratio of 11–22 and is a good fit for the standard sun given by ISO/TR 17801 [20]. In contrast, the HDRS procedure described in ISO 23698 uses the spectrum of ISO 24444:2019, which has a UVA to UVB ratio of 8 and is required to fulfil the narrow RCEE criteria. For all HDRS methods, the PMMA plates are irradiated with a dose that is calculated based on the UVA part of the irradiation spectrum and the UVA‐PF_DRS_. A systematic comparison of the photodegrading effect of the two spectra using UVA and UVB instable sunscreen samples would be helpful to better understand the influence of the different irradiation spectra on sunscreen products photo degradation. Both spectral definitions of ISO 24443 and ISO 24444 are expected to be appropriate for LED‐HDRS measurements with specific calibrations in the future.

(LED‐)HDRS seems to be a promising candidate for a non‐invasive SPF measurement that is sufficiently matching the UVA‐PF results of ISO 24443 and very close to matching the results of ISO 24444. It includes sunscreen product application on living skin and is therefore very close to the real situation of customers.

As described in the methodological paper by Colson et al. [14], it is also important to note that the criteria provide a framework for making decisions and failure to meet the performance criteria does not mean that a method should be disqualified. It is understood within ISO TC 217 WG 7 that criteria are evidence‐based and statistically sound elements for the experts to assess if a method displays sufficiently good performance and to consider if the standardization process can be completed (if initiated). [14]

The authors conclude that DRS measurements with a set of UVA‐LEDs instead of a Xenon arc lamp are an appropriate method as a third HDRS technology. Furthermore, the spectroscopic system used for this study further employed a fibre optic concept, detector, and in vitro irradiation spectrum which is different from the one described in ISO 23698.

The obtained re‐evaluation data of this work show that the method had similar performance to the gold standard methods ISO 24444 and ISO 24443 and that it can be proposed as an alternative method.

## CONFLICT OF INTEREST STATEMENT

G.W. and C.R. are employed by Courage + Khazaka electronic GmbH, which is the industrial partner with an interest in commercialization and owner of several patents.

## Supporting information


Appendix S1.

## Data Availability

The data that support the findings of this study are available from the corresponding author upon reasonable request.
