# Supplementary material for: Characterization of LED‐based hybrid diffuse reflectance spectroscopy method for determination of SPF and UVA‐PF in blinded multi‐centre study (ALT‐SPF)
Source: Int J Cosmet Sci. 2025 Sep 1;47(Suppl 1):96–114. doi: 10.1111/ics.70007 (PMC12400023; doi:10.1111/ics.70007)
Supplement: Supplementary file 1 — Appendix S1. [file ICS-47-96-s001.docx]

# Supplementary

Table 14: In vitro devices used in study 1 and study 2

|  | Lab 1 | Lab 2 | Lab 3 | Lab 4 |
| --- | --- | --- | --- | --- |
| ALT-SPF (study 1) | Customized, used e.g. in Ref. 18 | UV2000s labsphere | UV2000s labsphere | Custumized, used e.g. in Ref. 19 |
| Re-evalulation (study 2) | UV2000s labsphere | UV2000s labsphere | UV2000s labsphere | Custumized, used e.g. in Ref. 19 |
